# *In Silico* Insight into Potent of Anthocyanin Regulation of FKBP52 to Prevent Alzheimer's Disease

**DOI:** 10.1155/2014/450592

**Published:** 2014-05-12

**Authors:** Tzu-Chieh Hung, Tung-Ti Chang, Ming-Jen Fan, Cheng-Chun Lee, Calvin Yu-Chian Chen

**Affiliations:** ^1^Department of Biomedical Informatics, Asia University, Taichung 41354, Taiwan; ^2^School of Chinese Medicine, College of Chinese Medicine, China Medical University, Taichung 40402, Taiwan; ^3^Department of Biotechnology, Asia University, Taichung 41354, Taiwan; ^4^Department of Biological Science and Technology, China Medical University, Taichung 40402, Taiwan; ^5^School of Medicine, College of Medicine, China Medical University, Taichung 40402, Taiwan

## Abstract

Alzheimer's disease (AD) is caused by the hyperphosphorylation of Tau protein aggregation. FKBP52 (FK506 binding protein 52) has been found to inhibit Tau protein aggregation. This study found six different kinds of anthocyanins that have high binding potential. After analyzing the docking positions, hydrophobic interactions, and hydrogen bond interactions, several amino acids were identified that play important roles in protein and ligand interaction. The proteins' variation is described using eigenvectors and the distance between the amino acids during a molecular dynamics simulation (MD). This study investigates the three loops based around Glu85, Tyr113, and Lys121—all of which are important in inducing FKBP52 activation. By performing a molecular dynamic simulation process between unbound proteins and the protein complex with FK506, it was found that ligand targets that docked onto the FK1 domain will decrease the distance between Glu85/Tyr113 and Glu85/Lys121. The FKBP52 structure variation may induce FKBP52 activation and inhibit Tau protein aggregation. The results indicate that anthocyanins might change the conformation of FKBP52 during binding. In addition, the purple anthocyanins, such as cyanidin-3-glucoside and malvidin-3-glucoside, might be better than FK506 in regulating FKBP52 and treating Alzheimer's disease.

## 1. Introduction

Alzheimer's disease (AD) is an irreversible degenerative disease of the brain. As time passes, a patient's memory, language, intelligent judgment, and motor skills will gradually deteriorate. In 2010, about 36 million people had AD worldwide [1], and the medical expenses incurred totaled approximately $604 billion [2]. Huge medical expenses are associated with AD, and since AD almost always occurs in people over 65 years old, this disease becomes a great social burden in an aging society.

Drugs for Alzheimer's disease include cholinesterase inhibitors (such as Aricept (donepezil), Exelon (rivastigmine), and Reminyl (galantamine) [3]) and N-methyl D-aspartate (NMDA) antagonists Memantine (such as Witgen and Ebixa). These two categories [4–6] of drugs only slow down or ameliorate the symptoms but do not treat or prevent the disease [7]. Recent studies have found that the brains of AD patients appear to have protein aggregations that cause brain damage. Hyper-phosphorylated Tau protein aggregation is associated with AD and worsens the symptoms [8]. There are a lot of treatments based on gene, protein, enzyme, and pathway association with disease in recent years [9–14]. Thus, by expressing FKBP52, the aggregation of Tau protein could be inhibited [15], and such inhibition could form the basis for a treatment of Alzheimer's disease [16].

FKBP52 belongs to the FK506-binding protein family, which has a peptidyl prolyl isomerase (PPIase) functional domain. This domain will modify amino acids sites 231 and 255 of the Tau protein and make the Tau protein more readily phosphorylated [17]. An immunosuppressive drug containing FK506 could bind in this domain and inhibit PPIase activity [18]. FKBP52 could bind with steroid receptors in FKBPs [19]. FKBP52 contains four domains: a FKBP12 domain 1 (FK1), a FKBP12 domain 2 (FK2), a C-terminal tetratricopeptide repeat domain (TPR), and a calmodulin binding domain. The FK1 domain has a proline-rich loop, which is the PPIase activity domain. Therefore, the FK1 domain is the immunosuppressant binding site of FKBP52. Although the sequence of the FK2 domain is similar to the sequence of FK1, this domain lacks PPIase activity and cannot interact with FK506. The TPR domain helps FKBP52 bind heat-shock protein 90 (HSP90) as a cochaperone to remove Tau. Finally, the calmodulin-binding domain can regulate the phosphorylation of the protein [18, 20, 21].

Several studies have demonstrated that since the FK1 domain can bind PPIase, PPIase cannot modify Tau protein. Consequently, the calcineurin function will decrease, and Tau protein phosphorylation will be inhibited [22–27]. Furthermore, FKBP52 will have a higher binding affinity for HSP90 and steroid receptors (which could act as a cochaperone [28, 29]) than FKBP51, which has a similar structure to FKBPs and can make microtubules more stable [20, 30]. Therefore, this cochaperone can bring about Tau protein degradation [31, 32]. The ligand binds the FK1 domain, activates FKBP52, and reduces Tau protein phosphorylation while removing abnormal Tau proteins, thus preventing AD [18].

Anthocyanidins are a family of vegetable flavonoids and are the primary components in producing plant color and are well-known water-soluble dyes. The six common kinds of anthocyanidin are pelargonidin (red-orange), peonidin (red), delphinidin, cyanidin, petunidin, and malvidin (different shades of purple) [33–35]. This study used delphinidin-3-glucoside (D3G), petunidin-3-glucoside (Pt3G), cyanidin-3-glucoside (C3G), malvidin-3-glucoside (M3G), peonidin-3-glucoside (P3G), and pelargonidin-3-glucoside (Pa3G) as test compounds. In recent years, it has been found that anthocyanins can regulate immunity [36], have anticancer [37–40] and anti-inflammatory properties [41], as well as having preventative functions in cardiovascular disease [42–44] and diabetes [45, 46]. In addition, they are antioxidants [47–51], have skin brightening properties [52–55], can aid erection [56], and contain many other health benefits. The current literature indicates that the antioxidant capacity of anthocyanins can prevent the deterioration of beta-amyloid protein type AD [57–60].

The Computer-Aided Drug Design (CADD) is an* in silico* simulation technique containing structure-based and ligand-based simulation. The main aspects of structure-based simulation are molecular docking, and molecular dynamics. The protein-ligand interactions could be analyzed by the above technique [61–63].

In this study FK506, an efficacy drug [27, 64–68] with associated side effects [69–73], is used as a control drug. Our purpose is to determine whether anthocyanins influence FKBP52 activation, leading to the reduction of hyper-phosphorylated Tau protein aggregations, and thereby relieving Alzheimer's disease. To analyze the effects of the different anthocyanins on FKBP52 activation, we will observe the transformation of the FKBP52 structure after binding and molecular dynamic simulation.

Recently report, the personalized medicine and biomedicine are necessary [74, 75] especially in rare diseases [76] and diagnosis [77]. The TCM is a famous personalized medicine. In order to compare the effect on FKBP52 with the anthocyanins and the compounds of Traditional Chinese Medicine (TCM), we screened the TCM Database@Taiwan (http://tcm.cmu.edu.tw/) for simultaneous docking. The TCM Database@Taiwan [78] contains 61,000 TCM compounds and is the largest TCM database in the world. Recently, TCM database applied for stroke prevention [79], inflammation inhibition [80], pain regulation [81], cancer receptor inhibition [82, 83], and virus prevention [84, 85] by CADD and cloud-computing web server [86]. Thus, using CADD to analyze protein-ligand interaction is feasible in the research.

## 2. Materials and Methods

### 2.1. Data Collection

The FKBP52 protein structure was downloaded from the Protein Data Bank (PDB: 1Q1C) [87]. 1Q1C is the crystal structure of FKBP52 from amino acids 21 to 257. This structure includes the FK1 domain (amino acids 33 to 139) and the FK2 domain (amino acids 151 to 254). Current literature identifies the FK1 domain as the PPIase functional site and the FK506 binding site which is the FKBP52 activation site. Therefore, the FK1 domain is the binding site that detects the compounds of Traditional Chinese Medicine, by comparison with the control drug FK506. The six common anthocyanins are D3G, Pt3G, C3G, M3G, P3G, and Pa3G; their compounds and structures can be obtained from Pubchem [88].

### 2.2. Disorder Protein Detection

Disordered proteins are important in drug design, and thus protein structure and the ligand-interacting docking site should be detected [89, 90]. The protein sequence of CYP2C9 submitted to the Database of Protein Disorder (DisProt, http://www.disprot.org/) could predict the disordered region. Based on the result, the structure of docking site and drug efficiency could be discussed.

### 2.3. Docking

The control drug FK506 and anthocyanins (acting as ligands) were docked to the FK1 domain by LigandFit [91]. LigandFit, a program within Discovery Studio 2.5 (DS 2.5), is a receptor-rigid docking algorithm that uses a Monte Carlo simulation to measure the engaged position and orientation of the ligand when it targets the receptor of a crystal structure. The results were ranked based on docking score to assess the compatibility of the ligand and FKBP52 (1Q1C) crystal structure combination. If the ligand had a higher docking score than FK506, we could then use hydrophobic interaction analysis via Ligplot v.2.2.25 [92] to assess the interaction between ligand and protein amino acids.

### 2.4. Molecular Dynamics Simulation (MD)

Molecular dynamics simulation (MD) is a Discovery Studio 2.5 program and the protocol used is CHARMM force field [93] with minimization, heating, equilibration, and production stages. The interval time of each step was 2 fs in the force field. The Minimize stage utilized steepest descent [94] and conjugate gradient [95] to run the maximum 500 steps in two minimizations. Besides Minimizing, other stages were analyzed using the SHAKE algorithm. The system was heated from 50 K to 310 K gradually in the 50 ps heating intervals and then subjected to the 200 ps Balance period. Finally, a 20-nanosecond production period was used as a canonical ensemble—meaning that in all systems, *N*, *V* (volume) and *T* (temperature) were the same.

After obtaining results from the molecular dynamic simulation, the root mean square deviation (RMSD) of the protein-ligand complex and the value of total energy were calculated using Discovery Studio 2.5. We also used Discovery Studio 2.5 to detect the presence of hydrogen bonds between the protein and ligand (based on 2.5 Å distance) and calculated the torsion of the ligand structure during the molecular dynamics simulation. The H-bond occupancy was recorded in a table. OriginPro 8.5 used the RMSD, the value of total energy, and the torsion of ligand structure to analyze and draw diagrams.

The reference-identified eigenvector was used to represent the protein variation in protein interactions [96]. We calculated that the projection of the first two PCA (principal component analysis) eigenvectors would become the *X* and *Y* axes, based on the backbone of FKBP52's 256 amino acids of the MD data, to analyze the protein variation. The comparison between an unbound protein and a complex of protein with a ligand can describe the protein-ligand interaction.

Finally, we finished clustering based on the RMSD variation with a lapse of time in the molecular dynamic simulation and a threshold to distinguish the structure group of data. We identified the structure calculated in the intermediate period of the last population as the stable structure to determine that the interaction has been completed and balanced. The results of clustering can help analyze the variation of FKBP52 (1Q1C) structure under Docking, MD 0 ns and stability stages.

## 3. Results and Discussions 

### 3.1. The Detection of Disorder Protein

**Figure 1:**
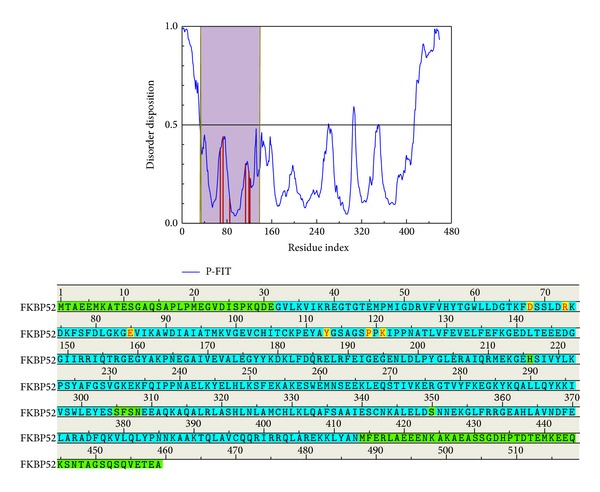
The disorder prediction and binding site detection. The blue curve in figure is the disorder disposition of each amino acid, the red lines indicate the residues of the important amino acids, and the purple region is the docking region, FK1 domain. The green regions of the amino-acid sequence show the predicted disordered regions, and the yellow regions, with the amino-acids noted in red mean important amino acids.

The disordered protein is an unstructured protein. The docking site consists of a disorder protein that will make the complex stabilize difficultly. The disordered regions are defined as the disposition greater than 0.5 (Figure 1). The purple region in Figure 1 is FK1 domain which has been defined functional site of FKBP52. This result indicates that the docking site and important amino acids do not consist of disorder protein. Thus, the disorder protein effect on our result is weak and the complex can stabilize.

### 3.2. Molecular Docking

The results show the six common anthocyanins and control drug FK506 docking to FKBP52 (Table 1) ranked from top to bottom based on the docking score. The docking scores for anthocyanins were about 1.5 to 2 times greater than those for FK506. This indicates that these ligands had a greater binding force than FK506 for the FK1 domain. We selected the results of screening the TCM database@Taiwan based on PLP1 and PLP2 being better than anthocyanin, and then ranked the docking score (Table 2). There were nine compounds over the threshold, even bisindolylpyrrole CPB-53-594-5 was better than the control in all conditions. Although we have these candidates, anthocyanin can be easily assimilated in the diet and does not have side effects.

**Figure 2:**
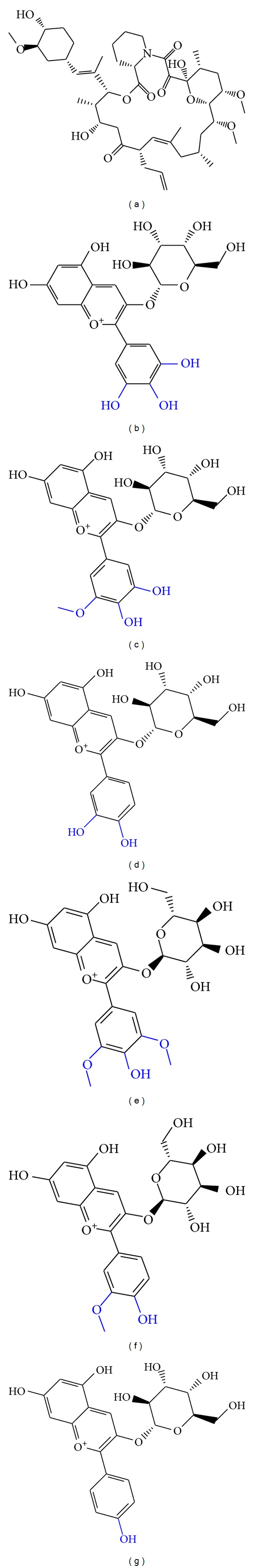
The structure of the ligand with (a) FK506 and (b) to (g) is D3G, Pt3G, C3G, M3G, P3G, and Pa3G, respectively, with the blue color indicating the differences.

**Figure 3:**
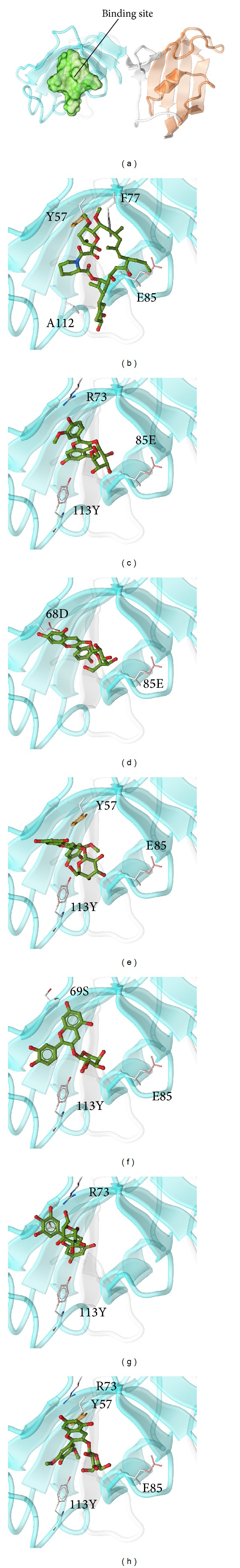
Docking poses of different ligands in the FKBP52 binding site. (a) Unbound protein, (b) FK506, (c) to (h) are D3G, Pt3G, C3G, M3G, P3G, and Pa3G, respectively.

Six anthocyanins as the ligands were arranged by differences in structure without taking into consideration the Cis and Trans isoforms which are shown in blue in Figure 2. Although their main structures are similar, the different branches ensure that their general structures do not overlap in the docking process (Figure 3). This result indicates that the ligands are not similar in docking with the same general structure and that it is helpful to analyze the interaction with different anthocyanins docking to FKBP52.

**Figure 4:**
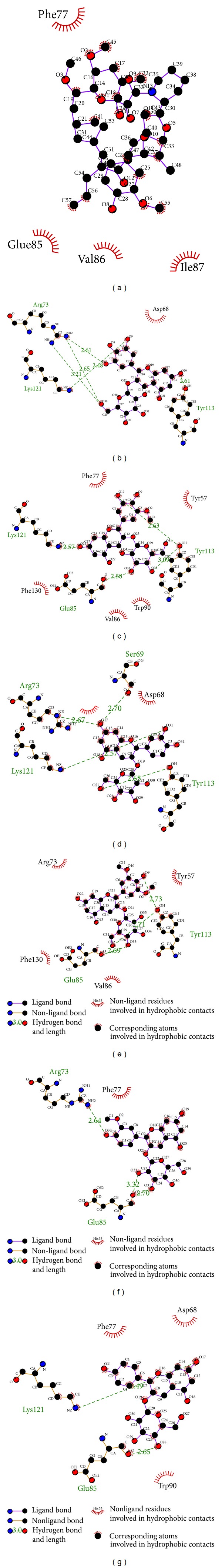
Ligplot illustrating protein-ligand interactions during docking. (a) FK506 and (b) to (g) indicate D3G, Pt3G, C3G, M3G, P3G, and Pa3G, respectively. Hydrophobic interactions are expressed by red spokes radiating towards the ligand atoms they contact in diagrams. Ligands are represented in purple. C, N, and O atoms are indicated in black, blue, and red, respectively.

Through ligplot v.2.2.25, Lys121, Tyr113, Glu85, and Arg73 were found to have a high percentage of H-bond and hydrophobic interactions (Figure 4), thus suggesting that they are important amino acids in FKBP52. The functional regions of FKBP52 [21, 87, 97–99] are identified as two loops containing Tyr113, Glu85, and Arg73 and are different between FK1 and FK2. This could determine whether PPIase functions or not, and the loop containing Lys121 will have an influence on calcineurin activity; therefore, these loops play an important role in ligand and protein interactions.

### 3.3. Molecular Dynamics

#### 3.3.1. RMSDs and Total Energy Trajectories

**Figure 5:**
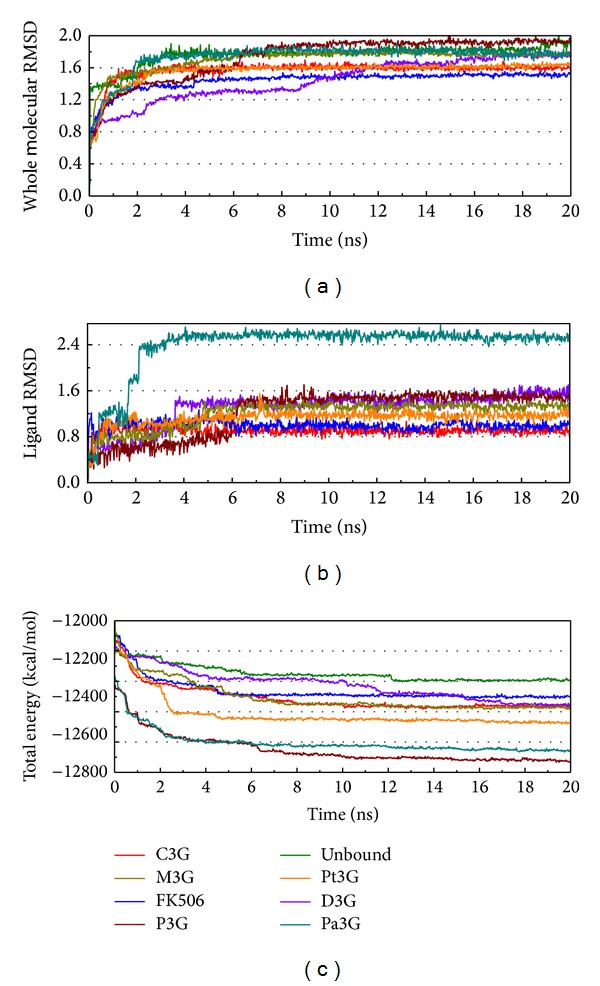
Measured trajectories during 20 ns MD. (a) Complex, (b) ligand, and (c) total energy during MD.

The data generated from molecular dynamics was analyzed for protein-ligand RMSD, ligand RMSD, and total energy of ligands-FKBP52 and unbound FKBP52 (Figure 5). This result shows that the total energy of unbound FKBP52 is the highest, with the complex of protein with FK506 being second, and with anthocyanins being the lowest. A lower total energy implies a more stable protein-ligand complex; this result indicates that anthocyanins bound to FKBP52 are more stable than FK506 or unbound proteins. In Figure 5, based on the gentle curve of the RMSD and the total energy, the low variation of protein-ligand interaction can be seen. This result shows that the interactions had been completed in 20 ns and that the data is credible for analysis.

**Figure 6:**
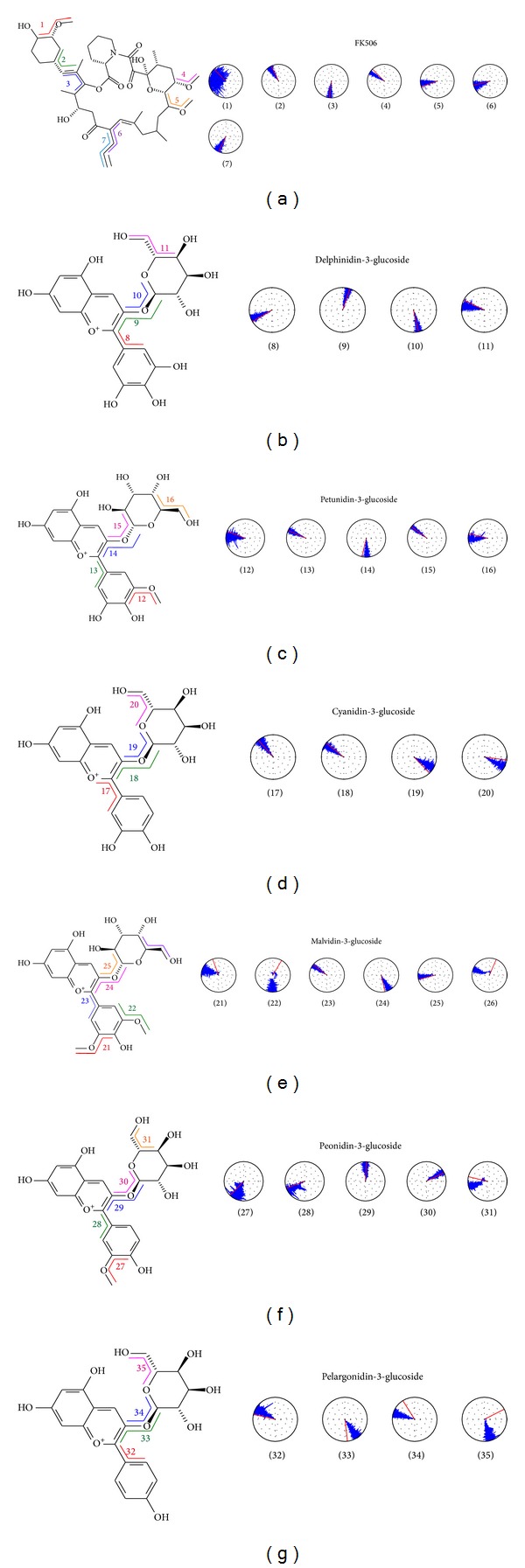
Torsion angles of anthocyanin during MD. Torsion angle measured is designated by the number which corresponds to the radar chart. The red and blue lines in the radar chart indicate the angle during time 0 and the period of MD.

The torsion in MD demonstrates that the base structure will set appropriate positions quickly, and thus interactions to change FKBP52 will only occur by a slight twist and offset.  Figure 6 presents the selected ligands that were found to be suitable in MD.

The H-bond plays an important role in protein and ligand interaction. We calculate the H-bond frequency of 1,000 interactions (0.02 ns is recorded as one interaction per 20 ns MD) while each ligand interacts with FKBP52 (Table 3). After analyzing the protein and ligand interactions from the docking process, the hydrophobic interactions, and the MD data, the results indicate that the amino acids Glu85, Tyr113, Lys121, Asp68, and Arg73 of FKBP52 formed many H-bonds during protein and ligand interaction (Table 4). After calculating the H-bond occupation of 7,000 interactions recorded from the seven ligands (FK506 and six anthocyanins), the amino acids with the three highest times of H-bond occupation were Glu85 (3099 times), Tyr113 (2357 times), and Lys121 (2135 times) in FKBP52. These occupations are obviously higher than the top four Asp68 (1794 times), top 5 Arg73 (1422 times), and others. It is suggested that Glu85, Tyr113, and Lys121 are important in FKBP52.

**Figure 7:**
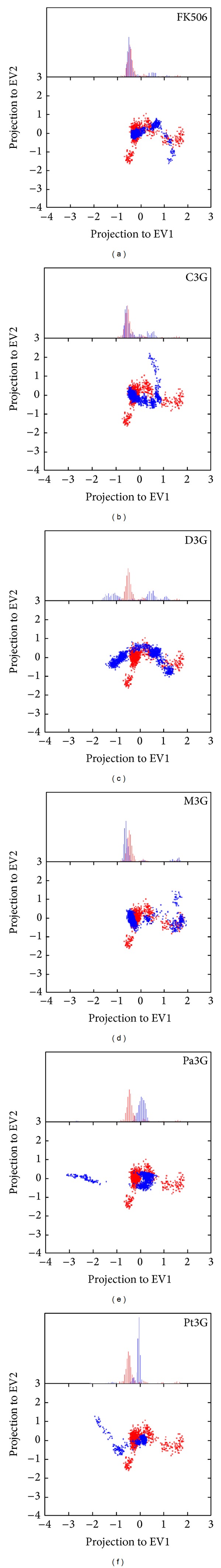
The PCA-eigenvector between ligand and unbound protein. The projection to the first two PCA-eigenvectors as *X*, *Y* axes based on the backbone of FKBP52 256 amino acids of MD is shown at the bottom of Figures 7(a) to 7(f). The red color indicates unbound protein and the blue is FKBP52 with ligand. The ligands in Figures 7(a) to 7(f) are FK506, C3G, D3G, M3G, Pa3G, and Pt3G.

According to the PCA-eigenvector of the FKBP52 backbone atoms of residues 21–257 (Figure 7) and the different distributions of the first eigenvector between control (blue) and unbound protein (red), FK506 is described as a ligand and made the first eigenvector distribution move left compared to unbound proteins, but their distributions are still similar. The results of C3G and M3G are similar to FK506. The direction of Pa3G and Pt3G first eigenvector distribution is from minus to plus and this direction is different from other ligands (which go from plus to minus). This shows that C3G and M3G may cause FKBP52 structural variations to be the same as FK506 when the protein and ligand interact. On the other hand, Pa3G and Pt3G may have different effects on FKBP52, especially out-lying data from the first eigenvector of 0–5 ns in MD.

**Figure 8:**
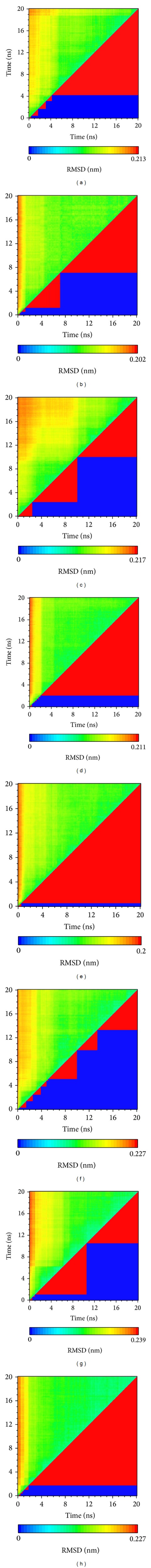
Clustering the ligand-protein interaction. (a) Unbound protein, (b) FK506, and (c) to (h) are D3G, Pt3G, C3G, M3G, P3G, and Pa3G, respectively. The mainly green triangle in upper left expresses the stability of the system, with the greater variation in red. The mainly red triangle in the lower right indicates groups calculated by RMSD variation during MD.

After protein and ligand interactions were finished, suitable protein structures were determined at 20 ns MD based on the curve of RMSD and the flattening of the total energy variation. After clustering, the data generated from MD in the same group indicate that their RMSD variation and structure are similar. The data generated from the calculations of the median of the last group period could be identified as stable structures (Figure 8). The results of clustering displaying C3G as ligand have the lowest RMSD variation and form the smallest group (only two groups) among unbound FKBP52 and seven ligands. One group of C3G and FKBP52 interaction in MD occurs in only the first twenty-eight out of 1000 data points, the others consist of the second group. This result indicates that if C3G performs as a ligand, FKBP52 will become stable in 0.58 ns and maintain the stability of the structure. Taking an analysis of clustering, the structures become stable in order of speed: C3G > (unbound protein) > Pt3G > Pa3G > D3G > FK506 > P3G > M3G.

Different FKBP52 stable structures in the docking process, at zero ns in MD, are observed, and this variation could be a result of protein and ligand interactions. The divergence of FKBP52 protein structure during MD between unbound protein and FK506 as a ligand was found to contain four loops in the FK1 domain, with each of them containing Arg73, Glu85, Tyr113, and Lys121, which would change their position during the MD period.

The result of ligplot and H-bond analysis shows that Glu85, Tyr113, and Lys121 had a more functional effect than the other amino-acids of FKBP52. Some references identify the two loops containing Glu85 and Tyr113 as the difference between the FK1 and FK2 domains, which function in PPIase immunosuppressive drug binding [21, 87, 97–99]. The loop with Lys121 has an influence on calcineurin activity, and the amino P119L of this loop is different in the FK1 domain between FKBP52 and FKBP51 [98]. Accordingly, it is feasible to describe the functional structure of FKBP52 by the distance variation of the three loops with Glu85, Tyr113, and Lys121 during docking, MD 0 ns, and stable stages.

**Figure 9:**
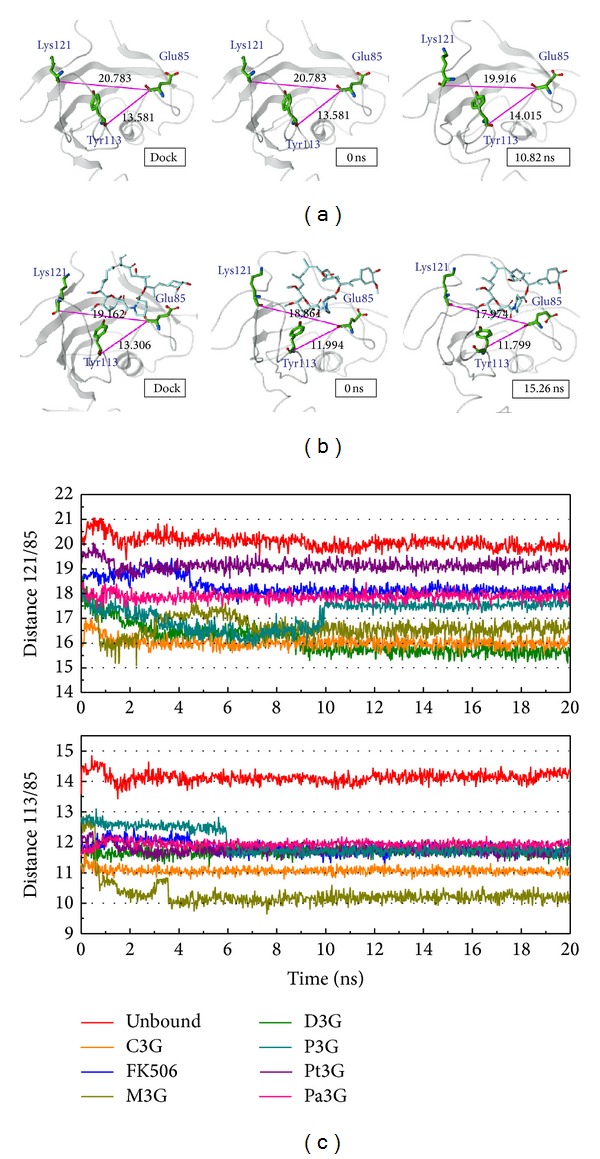
FKBP52 structure variation. (a) Unbound protein, (b) FK506, and (c) distance variation between unbound proteins with ligand.

There were obvious variations in the amino centroid positions of Glu85, Tyr113, and Lys121. Variations were found in the distance between Glu85/Tyr113 and Glu85/Lys121 but not between Tyr113/Lys121 (<1 Å). It was found that the distance between Glu85/Tyr113 increased from 13.581 Å to 14.015 Å, while the distance between Glu85/Lys121 shortened from 20.783 Å to 19.916 Å in unbound protein during three designed stages (Figure 9(a)). But in the case of FK506 as a ligand, the distances between Glu85/Tyr113 and Glu85/Lys121 decreased from 13.306 Å to 11.799 Å and from 19.162 Å to 17.974 Å (Figure 9(b)). From the differences from the unbound protein and FK506 as a ligand in MD, it is suggested that the ligands docking to the PPIase functional site will shorten Glu85/Tyr113 and Glu85/Lys121 during the three designed stages (Figure 9(c)).

**Figure 10:**
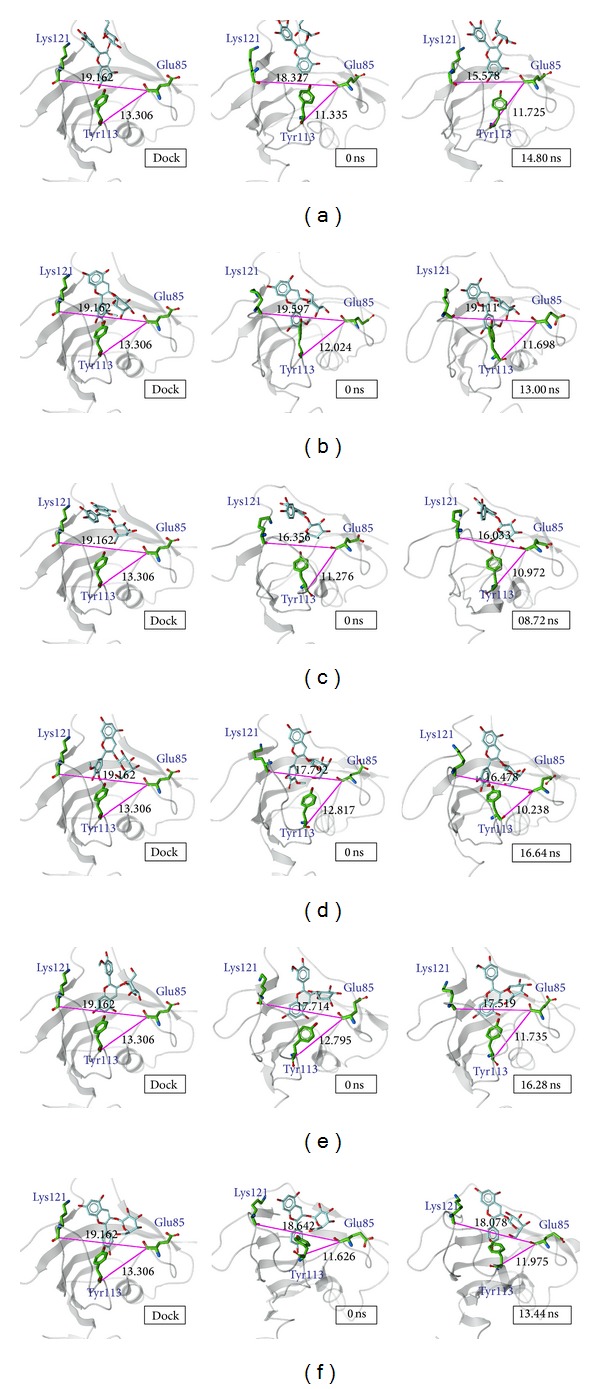
FKBP52 structure variation by anthocyanin target to FK1 domain. (a) D3G, (b) Pt3G, (c) C3G, (d) M3G, (e) P3G, and (f) Pa3G.

In Tables 5 and 6 we calculate these two distances from the docking, MD 0 ns, MD stable, and MD 20 ns. To compare the variation from 20 ns and stable protein structure of each condition (unbound, FK506, and anthocyanins), all the differences are less than the threshold. The above description presents the structure of the stable condition, with the 20 ns group being similar. The variations of Glu85/Tyr113 and Glu85/Lys121 are thought to dock to the PPIase functional site and activate FKBP52; these variations may provide a credible method of detection. We find the average variation of anthocyanin as a ligand shortened by about 2 Å. Glu85/Tyr113 and Glu85/Lys121 decreased from 13.306 Å to 11.391 Å and from 19.162 Å to 17.134 Å. These distances obviously shorten after anthocyanins dock to FK1, especially to D3G, C3G, and M3G. When D3G is a ligand, Glu85/Tyr113 and Glu85/Lys121 are −1.581 Å and −3.584 Å. The variation distances presented in the case of C3G are −2.334 Å and −3.129 Å and of M3G are −3.068 Å and −2.675 Å. The results of P3G and Pa3G were similar as FK506. Although Pt3G as a ligand had the smallest variation, the distances were still shorter than in the unbound protein (Figure 10). The above results illustrate that ligand docking to FK1 domain will affect Glu85/Tyr113 and Glu85/Lys121 explicitly, and anthocyanin could target PPIase functional site to activate FKBP52.

## 4. Conclusions

This research shows that the structure generated from the largest number of a final clustering group can become a stable condition for the final structure. The function of the PPIase inhibition and the FKBP52 activation can be suggested according to the variation of Glu85/Tyr113, and Glu85/Lys121 indicates the FKBP52 structural variation. Anthocyanins might regulate FKBP52 to prevent Alzheimer's disease based on the structure variation of FKBP52, especially the purple anthocyanins C3G and M3G. According to these results, these two anthocyanins could be predicted to have a better effect than the others. Due to their greater efficiency and fewer side effects, anthocyanins may become a more appropriate medicine than FK506.

## Figures and Tables

**Table 1:** Scoring functions of six anthocyanins and FK506 docking to FKBP52. The data is ranked by Dock Score.

Name	Dock Score	Binding Energy^#^	-PLP1	-PLP2
Delphinidin-3-glucoside	120.19	−199.264	31.6	44.69
Petunidin-3-glucoside	114.917	−160.598	62.24	71.86
Cyanidin-3-glucoside	112.451	−175.737	41.9	52.48
Malvidin-3-glucoside	111.318	−172.621	61.93	66.59
Peonidin-3-glucoside	105.531	−165.244	44.68	49.42
Pelargonidin-3-glucoside	94.527	−157.885	58.88	55.96
FK506*	**62.232**	**−202.882**	**58.04**	**50.16**

*Control.

^#^Unit: kcal/mol.

**Table 2:** Screening the TCM database docking to FKBP52 for results better than those for anthocyanin. The data are ranked by Dock Score.

Name	Herb	Dock Score	-PLP1	-PLP2
Saussureamine C	*Saussurea Iappa* CIarke	189.618	51.33	44.96
Chebulic acid	*PhyIIanthus urinaria* L	136.859	43.81	48.7
Bisindolylpyrrole-3^#^	*Lycogala epidendrum *	134.391	47.57	45.78
Nodifloridin A	*Lippia nodiflora* (L.) L. C. Rich.	133.342	45.56	55.25
Bisindolylpyrrole-5^#^	***Lycogala epidendrum***	**130.369**	**60.11**	**61.34**
Shogasulfonic acid A	*Celastrus paniculatus *	128.547	58.33	55.43
Tournefolic acid A	*Salvia miltiorrhiza *	124.716	45.92	50.98
Flazin	*Delphinium omeiense *	124.406	44.33	53.84
Phyllanthusiin E	*Melicope triphylla *	121.443	45.84	52.68
4-Gingesulfonic acid	*Celastrus paniculatus *	119.885	50.65	51.37
FK506*		**62.232**	**58.04**	**50.16**

*Control.

^#^The bisindolylpyrrole-3 is bisindolylpyrrole CPB-53-594-3, and bisindolylpyrrole-5 is bisindolylpyrrole CPB-53-594-5.

**Table 3:** H-bond occupancy for FKBP (1Q1C) with six kinds of anthocyanin and FK506 for a simulation time of 20 ns.

Name	H-bond interaction	Occupancy
FK506	Tyr113:HH/O9	3.70%
	Tyr57:HH/O1	61.30%
	Tyr57:HH/O2	0.30%

Delphinidin-3-glucoside	Asp72:OD1/H37	3.00%
	Asp68:OD1/H38	1.10%
	Asp68:OD2/H38	0.20%
	Glu85:O/H43	51.80%
	Arg73:HH12/O7	1.50%
	Arg73:HH21	2.30%
	Arg73:HH22/O7	99.40%
	Lys121:HZ/O9	28.20%
	Lys121:HZ2/O9	26.40%
	Lyd121:HZ3/O9	22.60%

Petunidin-3-glucoside	Asp68:OD1/H45	2.80%
	Phe67:O/H45	2.30%
	Glu85:O/H56	100.00%
	Arg73:HH21/O22	1.10%
	Lys121:HZ1/O21	35.00%
	Lys121:HZ2/O21	43.90%
	Lys121:HZ3/O21	46.30%
	Trp90:HE1/O9	92.60%
	Tyr113:HH/O29	1.90%
	Tyr113:HH /O32	98.00%

Cyanidin-3-glucoside	Ser69:O/H39	97.80%
	Tyr113:OH/H48	2.30%
	Glu85:O/H49	55.20%
	Pro120:O/H53	93.30%
	Arg73:HE/O17	0.10%
	Arg73:HH11/O17	16.40%
	Tyr113:HH/O27	0.40%
	Tyr57:HH/O27	2.40%

Malvidin-3-glucoside	Tyr113:OH/H41	0.60%
	Tyr113:OH/H58	5.50%
	Glu85:O/H58	0.10%
	Arg73:HH12/O22	0.30%
	Arg73:HH21/O22	7.40%
	Arg73:HH22/O22	0.10%
	Tyr113:HH/O10	4.30%
	Tyr113:HH/O32	1.10%

Peonidin-3-glucoside	Tyr113:OH/H44	59.3%
	Val86:O/H44	60.2%
	Tyr113:OH/H52	57.7%
	Val86:O/H52	4.1%
	Glu85:O/H53	95.8%
	Glu85:O/H54	2.3%
	Arg73:HH12/O2	0.30%
	Tyr113:HH/O29	0.30%
	Tyr57:HH/O18	0.10%

Pelargonidin-3-glucoside	Asp68:OD1/H39	89.40%
	Asp68:OD2/H39	85.90%
	Tyr113:OH/H48	0.10%
	Glu85:O/H49	4.40%
	Arg73:HH21/O17	0.50%
	Arg73:HH22/O17	13.10%
	Lys121:HZ1/O17	1.70%
	Lys121:HZ2 /O17	8.40%
	Lys121:HZ3/O17	1.00%
	Tyr113:HH/O27	0.50%
	Tyr57:HH/O19	0.10%

H-bond occupancy cutoff: 2.5 Å.

**Table 4:** Summary of interaction type, location, and frequency of test ligands following docking and MD simulation.

Interaction location/type/frequency*										
Ligand	Glu85	Tyr113	Lys121	Asp68	Arg73	Ser69	Pro120	Trp90	Val86	Tyr57
FK506 docking										
FK506 ligplot	Y								Y	
FK506 MD		H								HH

D3G docking		H	H		HH					
D3G ligplot		H	H	Y	H					
D3G MD	H		HHH	HH	HHH					

Pt3G docking	H	H	H							
Pt3G ligplot	H	H	H					Y	Y	Y
Pt3G MD	H	HH	HHH	H	H			H		

C3G docking		H	H		H*π*	H				
C3G ligplot		H	H	Y	H	H				
C3G MD	H	HH			HH	H	H			H

M3G docking	H	HH			H					*π*
M3G ligplot	H	H			Y				Y	Y
M3G MD	H	HHHH			HHH					

P3G docking	H	H			H					
P3G ligplot	H				H					
P3G MD	HH	HHH			H				HH	H

Pa3G docking	H		HH	H						
Pa3G ligplot	H		H	Y				Y		
Pa3G MD	H	HH	HHH	HH	HH					H

*Each letter denotes one interaction.

*π*: pi-interaction.

Y: Hydrophobic interaction.

H: H-bond.

**Table 5:** Comparing amino distance variation from Lys121 to Glu85 while the protein is in an unbound condition and targets the ligand during docking and MD.

	Docking	0 ns	Stable	20 ns
	121/85	121/85	121/85	121/85
Unbound	20.783	20.783	19.916	20.215
		(0)	(−0.867)	(−0.568)
506	19.162	18.861	17.974	17.990
		(−0.301)	(−1.188)	(−1.172)
D3G	19.162	18.327	15.578	15.450
		(−0.835)	(−3.584)	(−3.712)
Pt3G	19.162	19.597	19.111	19.033
		(0.435)	(−0.051)	(−0.129)
C3G	19.162	16.356	16.033	16.199
		(−2.806)	(−3.129)	(−2.963)
M3G	19.162	17.792	16.487	16.567
		(−1.37)	(−2.675)	(−2.595)
P3G	19.162	17.714	17.519	17.587
		(−1.448)	(−1.643)	(−1.575)
Pa3G	19.162	18.642	18.078	18.102
		(−0.52)	(−1.084)	(−1.060)
Anthocyanin average	19.162	18.071	17.134	17.156
		(−1.091)	(−2.028)	(−2.006)

**Table 6:** Comparing amino-acid distance variation from Tyr113 to Glu85 while the protein is in an unbound condition and targets the ligand during docking and MD.

	Docking	0 ns	Stable	20 ns
	113/85	113/85	113/85	113/85
Unbound	13.581	13.581	14.015	14.398
		(0)	(0.43)	(0.817)
506	13.306	11.994	11.799	11.690
		(−1.312)	(−1.507)	(−1.616)
D3G	13.306	11.335	11.725	11.626
		(−1.971)	(−1.581)	(−1.680)
Pt3G	13.306	12.024	11.698	11.522
		(−1.282)	(−1.608)	(−1.784)
C3G	13.306	11.276	10.972	10.945
		(−2.03)	(−2.334)	(−2.361)
M3G	13.306	12.817	10.238	10.041
		(−0.489)	(−3.068)	(−3.265)
P3G	13.306	12.795	11.735	11.891
		(−0.511)	(−1.571)	(−1.415)
Pa3G	13.306	11.626	11.975	11.897
		(−1.68)	(−1.331)	(−1.409)
Anthocyanin average	13.306	11.979	11.391	11.320
		(−1.327)	(−1.915)	(−1.986)
